# Risk of all-cause and CHD mortality in women versus men with type 2 diabetes: a systematic review and meta-analysis

**DOI:** 10.1530/EJE-18-0792

**Published:** 2019-01-21

**Authors:** Guodong Xu, Dingyun You, Liping Wong, Donghui Duan, Fanqian Kong, Xiaohong Zhang, Jinshun Zhao, Wenhua Xing, Li Li, Liyuan Han

**Affiliations:** 1 Department of Epidemiology, Zhejiang Provincial Key Laboratory of Pathophysiology, School of Medicine, Ningbo University, Ningbo; 2 Department of Science and Technology, Kunming Medical University, Kunming, China; 3 Department of Social and Preventive Medicine, Faculty of Medicine, University of Malaya, Kuala Lumpur, Malaysia; 4 Department of Endocrinology and Metabolism, Ningbo First Hospital, Ningbo, Zhejiang, China

## Abstract

**Objective:**

Previous studies have shown sex-specific differences in all-cause and CHD mortality in type 2 diabetes. We performed a systematic review and meta-analysis to provide a global picture of the estimated influence of type 2 diabetes on the risk of all-cause and CHD mortality in women vs men.

**Methods:**

We systematically searched PubMed, EMBASE and Web of Science for studies published from their starting dates to Aug 7, 2018. The sex-specific hazard ratios (HRs) and their pooled ratio (women vs men) of all-cause and CHD mortality associated with type 2 diabetes were obtained through an inverse variance-weighted random-effects meta-analysis. Subgroup analyses were used to explore the potential sources of heterogeneity.

**Results:**

The 35 analyzed prospective cohort studies included 2 314 292 individuals, among whom 254 038 all-cause deaths occurred. The pooled women vs men ratio of the HRs for all-cause and CHD mortality were 1.17 (95% CI: 1.12–1.23, *I*^2^ = 81.6%) and 1.97 (95% CI: 1.49–2.61, *I*^2^ = 86.4%), respectively. The pooled estimate of the HR for all-cause mortality was approximately 1.30 in articles in which the duration of follow-up was longer than 10 years and 1.10 in articles in which the duration of follow-up was less than 10 years. The pooled HRs for all-cause mortality in patients with type 2 diabetes was 2.33 (95% CI: 2.02–2.69) in women and 1.91 (95% CI: 1.72–2.12) in men, compared with their healthy counterparts.

**Conclusions:**

The effect of diabetes on all-cause and CHD mortality is approximately 17 and 97% greater, respectively, for women than for men.

## Introduction

Diabetes is recognized as the world’s fastest growing chronic condition. Due to rapid increases in the prevalence of physical inactivity, overweight and obesity, the number of people with diabetes is projected to rise to 592 million by 2035 (1). In particular, type 2 diabetes (T2D) has attained the status of a global pandemic, with the total number of patients with T2D estimated at 425 million in 2015 (2).

Accumulating evidence documents T2D as an independent risk factor for all-cause mortality (3, 4, 5, 6). The risk of all-cause mortality in persons with T2D is approximately doubled (7). However, these conclusions are mainly based on the assumption that the risk of diabetes in women is the same as in men (8). However, evidence is accruing that the detrimental effects of diabetes are higher among women than among men (9). The sex-based difference in the risk of diabetes would not only result from patient management and treatment (10) but also from the diversity of biological factors (11). Epidemiological studies reported that women with T2D had a higher risk of mortality from cardiovascular diseases (10, 12) and cancer (13) than men.

However, the differences between men and women in the risk of all-cause and CHD mortality is still unclear. Moreover, none of the relevant meta-analyses summarized the differences in risk of all-cause and CHD mortality between men and women. Therefore, we performed a comprehensive meta-analysis to estimate reliably the effect of T2D on all-cause and CHD risk among women in comparison with men.

## Methods

### Search strategy

We systematically searched the PubMed (www.ncbi.nlm.nih.gov), EMBASE and Web of Science databases (from their starting dates to Aug 7, 2018) with the limitations object human and language English. A combined text word and medical subject heading (MeSH) search strategy was applied with the terms ‘mortality’, ‘death’, ‘Diabetes Mellitus, Type 2’, ‘Adult-Onset Diabetes’, ‘Non-Insulin-Dependent Diabetes’, ‘Gender’, ‘Sex’, ‘Cohort’, ‘Prospective’ and ‘Longitudinal’. We also scanned the reference lists of relevant reviews and meta-analyses to discern additional potentially relevant literature.

### Inclusion and exclusion criteria

We included articles only when they had clearly reported hazard ratios (HRs) or equivalents for all-cause or CHD mortality in both genders (T2D patients vs healthy counterparts). We also included articles that did not report HRs for each gender directly, but from which we could calculate it. Studies in which the enrolled participants had stroke, coronary heart disease or other cardiovascular diseases (myocardial infarction, atherosclerosis etc.) were excluded. If more than two articles had been published about the same cohort, we enrolled the one with the longer follow-up period or a larger sample size. The search strategy and inclusion criteria were defined and agreed upon by all the authors. The quality of the included studies was evaluated by the NOS (Newcastle-Ottawa Scale) (14) (Supplementary data, see section on supplementary data given at the end of this article). Our meta-analysis was performed in accordance with the PRISMA statement (15) and registered at the International Prospective Register of Systematic Reviews (Prospero) (http://www.crd.york.ac.uk /PROSPERO, registration number: CRD42017074187).

### Data extraction

For each study, we extracted the following variables: name of first author and study, baseline years of study, country of study, duration of follow-up, mean ages of participants, sample size, death count, adjusted variables, HRs and their 95% CIs in men and women and NOS score. Two authors (Guodong Xu and Dingyun You) independently extracted the data. If there was controversy, the discrepancy was resolved by an arbitrator (Liyuan Han).

### Statistical analysis

We extracted gender-specific HRs and 95% CIs from each study (T2D patients vs healthy counterparts). Subsequently, gender-specific HRs and 95% CIs were used to estimate the pooled ratio of HR and the corresponding 95% CIs. Subgroup analyses were performed by year of study baseline (before 1980, 1980 to 1990 and after 1990); region (America, Europe, Asia, and Australia, Canada, New Zealand or Pacific); duration of study (<10, 10–14, >14 years); study quality (NOS score) (≥6 vs <6) (14) and adjusted status (unadjusted vs adjusted). Sensitivity analysis was conducted to ascertain the stability of the pooled results after removing one study at a time. The *I* ^2^ value was used to estimate heterogeneity. An *I* ^2^ value of 25, 50 and 75% represented a low, middle and high degree of heterogeneity, respectively (16). Meta-regression analyses were also performed to estimate the source of heterogeneity. We used funnel plots to estimate publication bias. Egger’s and Begg’s test were also applied to quantitatively estimate publication bias. Additionally, to explore the possible effect of publication bias, we employed trim-and-fill method (17) in our meta-analyses for more reliable estimates. All *P* values were two sided and *P* values less than 0.05 were considered as statistically significant. Software Stata 12.0 (StataCorp) was used to perform statistical analyses.

## Results

### Study characteristics

We systematically searched PubMed, EMBASE and Web of Science (from their starting dates to Aug 7, 2018). A total of 3907 articles were identified by assessment of titles and abstracts, and eight additional records were identified from the reference lists (Supplementary Fig. 1). After full-text assessment, 35 articles were finally included in our meta-analysis (18, 19, 20, 21, 22, 23, 24, 25, 26, 27, 28, 29, 30, 31, 32, 33, 34, 35, 36, 37, 38, 39, 40, 41, 42, 43, 44, 45, 46, 47, 48, 49, 50, 51, 52) for all-cause mortality, and 24 articles for CHD mortality (21, 25, 26, 29, 30, 40, 43, 47, 53, 54, 55, 56, 57, 58, 59, 60, 61, 62, 63, 64, 65, 66). Table 1 shows the baseline characteristics of all 52 cohorts. A total of 2 314 292 T2D cases (46% women) were included, and 254 038 all-cause deaths (45% women) occurred. Among the 35 datasets included, 23 cohort studies were performed in Europe (19, 20, 24, 25, 27, 28, 29, 30, 31, 32, 33, 34, 36, 37, 38, 39, 41, 42, 43, 45, 48, 49, 51), 5 in Australia, Canada, New Zealand or Pacific (22, 26, 35, 44, 46), 3 in America (18, 21, 22) and 6 in Asia (23, 26, 40, 47, 50, 52). All the included articles had NOS scores higher than four points, and 26 of them scored at least six points (18, 21, 23, 25, 26, 28, 29, 31, 32, 33, 35, 36, 38, 39, 40, 41, 43, 44, 46, 47, 48, 49, 50, 51, 52).

**Table 1:** Characteristics of included studies.

	Baseline years	Country	Follow-up duration (years)	Participants (*n*)	% women	Mean age (years)	Deaths (*n*)	% deaths in women	Ascertainment of diabetes	Variables used to standardize HR	Causes of death	NOS score
Framingham Heart Study (earlier) (19)	1950–1975	US	25	399	52	56.7	109	45	Self-reported, Measured	Age, sex	All-cause	7
Bedford Diabetes Study (19)	1962–1971	UK	10	363	50	NA	108	56	Measured	Age	All-cause	5
Reykjavik Diabetes Study (20)	1967–1991	Iceland	17	477	44	55	213	31	Measured	NA	All-cause	5
NHANES I (21)	1971–1984	US	14	407	54	62.5	172	41	NA	Age, smoking, SBP, TC, BMI	All-cause, CHD	6
WHO MSVDD (22)	1975–1979	US	15	240	46	NA	51	35	NA	Age	All-cause	5
GRIC (23)	1975–1984	India	10	1266	58	NA	241	47	Self-reported, Measured	Age	All-cause	6
Framingham Heart Study (later) (18)	1975–2005	US	25	679	NA	59.5	159	31	Self-reported, Measured	Age, sex	All-cause	7
WHO MSVDD (Switzerland) (24)	1977–2006	Switzerland	30	308	44	48.6	214	43	Self-reported	NA	All-cause	5
Swedish Annual Level-of-Living Survey (25)	1979–1985	Sweden	7	776	46	NA	418	46	Self-reported	Age	All-cause, CHD	6
Diabetes Melanesian Fijians Cohort Study (26)	1980–1991	Melanesian	11	65	65	NA	25	48	Measured	Age	All-cause, CHD	6
Diabetes Asian Indian Cohort Study (26)	1980–1991	India	11	166	52	NA	21	34	Measured	Age	All-cause, CHD	6
Denmark Diabetes Register (27)	1981–1993	Denmark	13	228	60	68	75	48	Measured	NA	All-cause	5
Kuopio Diabetes Register (28)	1981–1995	Finland	15	133	47	55.7	59	47	Measured	Age	All-cause	6
Finland Diabetes Study (29)	1982–1997	Finland	17	962	48	42.7	399	48	Self-reported, Measured	Age, education years, BMI, SBP, TC, and smoking	All-cause, CHD	7
Diabetes Finland Cohort Study (30)	1982–2001	Finland	18	1059	45	58.1	768	45	Measured	NA	All-cause, CHD	5
Poole Diabetes Registry (31)	1983–1991	UK	8	917	48	60.8	295	47	Self-reported, Measured	NA	All-cause	6
DISS (32)	1983–1992	Sweden	10	661	NA	NA	14	21	Self-reported, Measured	Age	All-cause	6
DISS (33)	1983–1999	Sweden	9	1142	NA	NA	37	22	Measured	Age, sex	All-cause	7
Verona Diabetes Study (34)	1987–1991	Italy	5	7148	53	NA	1550	52	Measured	NA	All-cause	5
Prospective Dubbo Study of Australian (35)	1988–1993	Australian	5	207	49	69.9	61	39	Self-reported, Measured	NA	All-cause	6
Diabetes New Zealand Cohort Study (36)	1989–1999	New Zealand	10	447	53	62	187	54	Self-reported, Measured	Age, sex	All-cause	7
FRESCO (37)	1991–2005	Spanish	10	8627	47	60.9	781	44	Self-reported	NA	All-cause	5
Diabetes Spain Cohort Study (38)	1991–2006	Spain	9	469	54	60.4	80	40	Measured	Age, HDL, smoking	All-cause	6
Norwegian Diabetes Register (39)	1991–1999	Italy	9	29 656	NA	NA	6673	48	Measured	Age, area of birth	All-cause	6
Takayama Diabetes Study (40)	1992–1999	Japan	8	1217	65	60.1	176	36	Measured	Age, smoking, BMI, physical activity, education years, hypertension, total energy intake, intake of vegetables, fat, and alcohol	All-cause, CHD	7
GPRD (41)	1992–1999	UK	8	44 230	46	67	12 453	76	Measured	Age, sex	All-cause	7
Record-linkage Databases (42)	1993–2004	UK	12	10 532	48	NA	1863	47	Measured	NA	All-cause	5
South Tees Diabetes Mortality Study (43)	1994–1999	UK	6	4081	45	NA	1151	45	Measured	Age, sex, calendar year	All-cause, CHD	7
CCDSS (44)	1995–2008	Canada	10	15 152	19	59.9	3554	48	Measured	Region of residence, socioeconomic status quintile,	All-cause	6
Diabetes Clinic of the San Giovanni Battista Hospital (45)	1996–2000	Italy	5	2673	NA	70	428	46	Self-reported, Measured	NA	All-cause	4
National Diabetes Services Scheme (1997–03) (46)	1997–2003	Australia	7	10 60367	46	60	76 689	43	Self-reported, Measured	Age, sex	All-cause	7
ET-CHD Registry (47)	1997–2006	China	10	386	38	64.6	157	42	Self-reported, Measured	Age, smoking status, HDL, TC, creatinine, stroke, cancer	All-cause, CHD	6
National Diabetes Registry (48)	1998–2003	UK	5	736	45	64.2	147	52	Self-reported, Measured	NA	All-cause	6
University Hospital Birmingham (49)	2000–2007	UK	7	679	36	NA	100	42	Measured	Age	All-cause	6
NHISNSC (50)	2002–2004	Korean	3	29 807	48	NA	7103	44	Self-reported, Measured	Age, sex	All-cause	6
National Diabetes Services Scheme (2004–10) (46)	2003–2010	Australia	7	1 060 367	46	NA	134 393	44	Self-reported, Measured	Age, sex	All-cause	7
GPRD (51)	2004–2010	UK	7	21 789	40	55.1	2146	35	Measured	Age, sex, and general practice	All-cause	7
DIAMOND Cohort Registry (52)	2010–2012	Korean	2	1125	34	64.9	44	52	Self-reported, Measured	NA	All-cause	6
Tecumseh Study (59)	1959–1979	US	6	386	60	NA	230	55	Measured	Age, BMI, smoking, use of hypertension medications	CHD	6
The Reykjavik Study (60)	1961–1991	Iceland	9	547	31	NA	50	12	Measured	Age, TC, SBP, ECG, education	CHD	6
Chicago Heart Association Detection Project (61)	1967–1973	US	19	5729	46	51.4	183	41	Self-reported, Measured	Age, education, smoking, alcohol intake, physical activity, BMI, hypertension, diabetes	CHD	7
NHANES I (62)	1971–1992	US	20	462	62	59.2	127	54	NA	Age, smoking, hypertension, TC, BMI	CHD	5
Framingham Heart Study (63)	1971–1995	US	20	178	42	NA	35	46	Measured	Age, hypertension, TC, BMI, smoking	CHD	6
The Rancho Bernardo Study (64)	1972–1985	US	14	334	38	63.4	55	35	Self-reported, Measured	Age	CHD	7
Hawaii-Los Angeles-Hiroshima Study (65)	1976–1984	Hawaii	7	776	46	64.5	183	48	Self-reported, Measured	Age	CHD	6
The Adventist Health Study (66)	1977–1982	US	6	812	68	NA	33	61	Self-reported	Age	CHD	5
Community-dwelling Elderly (53)	1982–1988	US	11	166	52	NA	12	42	Measured	Age, study year, smoking, TC, HDL, SBP, BMI	CHD	6
Finland Diabetes Study (54)	1982–1990	Finland	20	113	62	NA	36	47	NA	Age, sex	CHD	7
Finland National Hospital Discharge Register (55)	1982–1994	Finland	12	14 786	52	NA	294	21	Self-reported, Measured	Age, TC, TG, BMI, hypertension, smoking	CHD	4
Finnish Diabetes Study (56)	1986–1988	Finnish	10	386	38	NA	105	50	Measured	Age	CHD	5
NHANES III (57)	1988–2006	US	12	133	56	NA	23	61	Measured	Age, BMI, UA, TC, TG, hypertension, smoking	CHD	7
JACC Study (58)	1988–2009	Japan	8	1217	35	NA	15	13	Measured	Age, race, education, BMI, smoking, SBP, DBP, HDL, medication use,	CHD	7
GeneSTAR (57)	1993–2005	US	7	292	48	46.5	17	41	NA	Age	CHD	6
MESA (57)	2000–2011	US	14	407	54	52.6	59	36	NA	Age	CHD	7

*Including UK, Switzerland, Poland, Germany, Croatia, China, Japan, Cuba, USA.

BMI, body mass index; CCDSS, Canadian Chronic Diseases: Surveillance System; DISS, Diabetes Incidence Study in Sweden; ET-CHD, Eastern Taiwan integrated health care delivery system of Coronary Heart Disease; FRESCO, Función de Riesgo ESpañola de acontecimientos Coronarios y Otros; GPRD, General Practice Research Database; GRIC, Gila River Indian Community; HDL, high density lipoprotein; HR, hazard ratio; JACC, The Japan Collaborative Cohort Study; MESA, Multi-Ethnic Study of Atherosclerosis; MSVDD, Multinational Study of Vascular Disease in Disease in Diabetes; NA, not available; NHANES, the National Health and Nutrition Examination Survey; NHISNSC, National Health Insurance ServiceNational Sample Cohort; TC, total cholesterol.

### HRs for all-cause and CHD mortality between men and women

**Figure 1:**
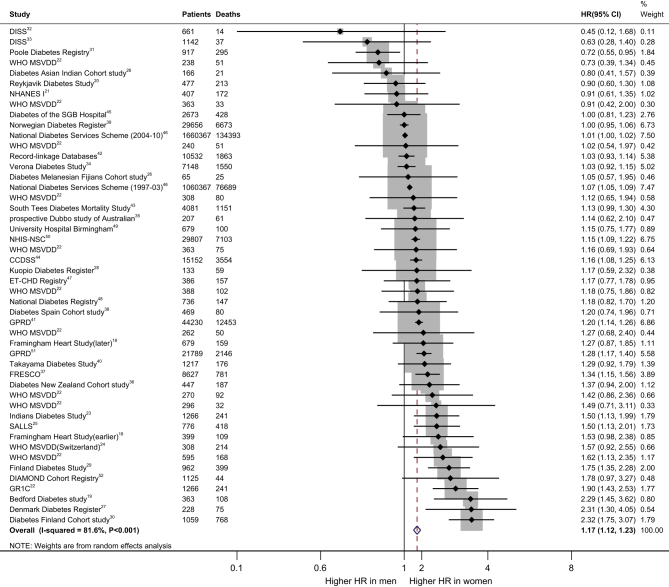
Pooled women-to-men ratios of HRs for all-cause mortality, comparing people with type 2 diabetes vs those without the disorder. CCDSS, Canadian Chronic Diseases: Surveillance System; DISS, Diabetes Incidence Study in Sweden; ET-CHD, Eastern Taiwan integrated health care delivery system of Coronary Heart Disease; FRESCO, Función de Riesgo ESpañola de acontecimientos Coronarios y Otros; GPRD, General Practice Research Database; GR1C, Gila River Indian Community; HR, hazard ratio; MSVDD, Multinational Study of Vascular Disease in Disease in Diabetes; NHANES, the National Health and Nutrition Examination Survey; NHISNSC, National Health Insurance Service‑National Sample Cohort.

**Figure 2:**
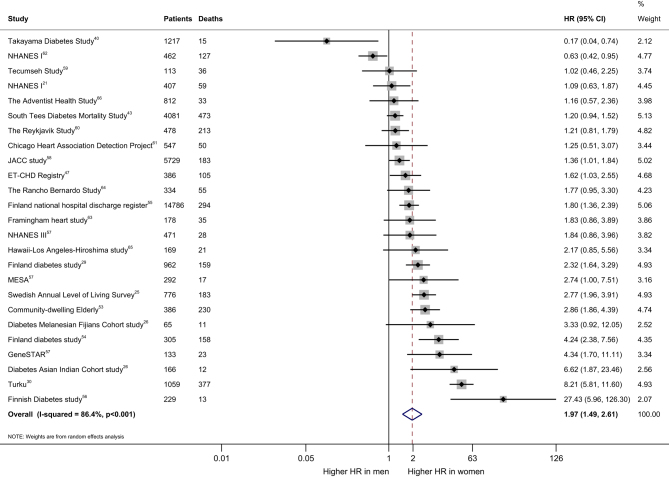
Pooled women-to-men ratios of HRs for CHD mortality, comparing people with type 2 diabetes vs those without the disorder. ET-CHD, Eastern Taiwan integrated health care delivery system of Coronary Heart Disease; HR, hazard ratio; JACC, The Japan Collaborative Cohort Study; MESA, Multi-Ethnic Study of Atherosclerosis; NHANES, the National Health and Nutrition Examination Survey.

The effect of diabetes on all-cause mortality is 17% higher in women than men (HR 1.17. (95% CI: 1.12–1.23)) (Fig. 1). The pooled women vs men HR for CHD mortality was 1.97 (95% CI: 1.49–2.61) (Fig. 2). However, the *I* ^2^ value of 81.6 and 86.4%, respectively, implying the possibility of significant heterogeneity between studies. The pooled HR for all-cause mortality in patients with T2D was 2.33 (95% CI: 2.20–2.69) in women (Supplementary Fig. 3) and 1.91 (95% CI: 1.72–2.12) in men (Supplementary Fig. 4), when compared with their healthy counterparts. The pooled HR for CHD mortality in patients with T2D was 3.79 (95% CI: 3.01–4.78) in women (Supplementary Fig. 5) and 2.13 (95% CI: 1.86–2.44) in men (Supplementary Fig. 6), when compared with their healthy counterparts.

### Subgroup analysis

**Figure 3:**
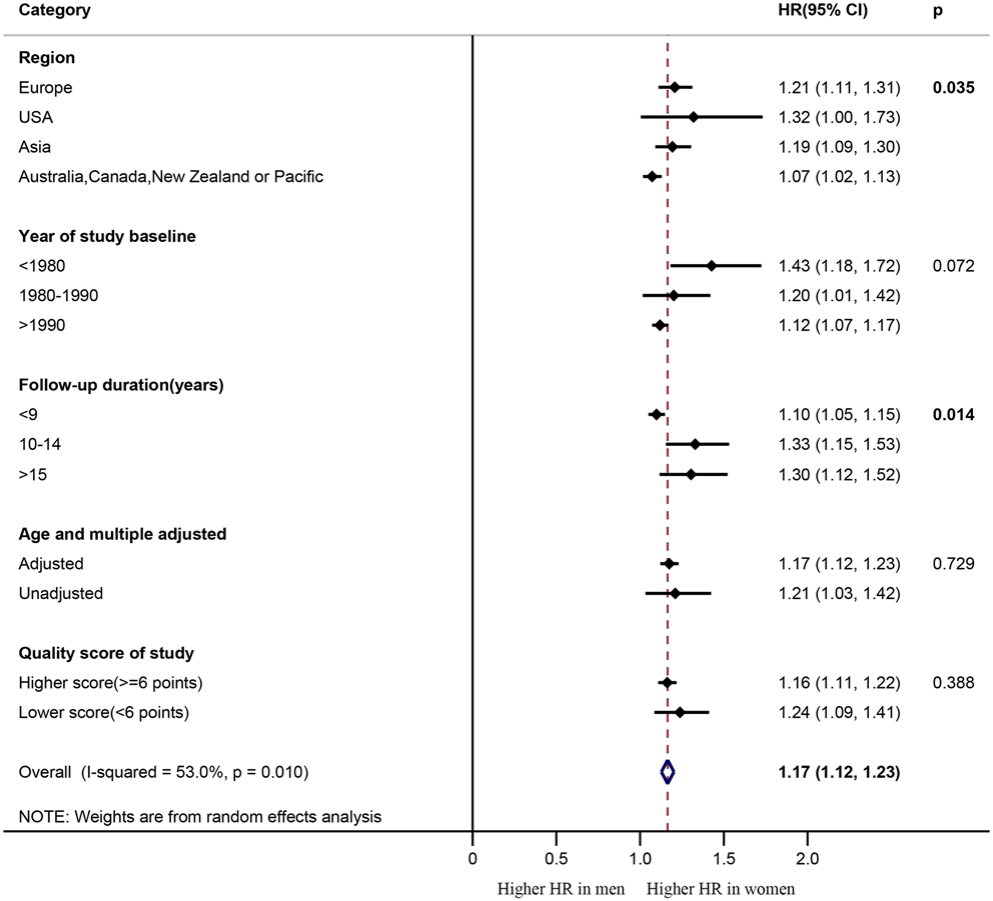
Forest plot of the subgroup analyses with pooled women-to-men ratios of HRs for all-cause mortality. HR, hazard ratio.

We used subgroup analysis to explore the sources of heterogeneity. The year in which the study began did not explain the possible heterogeneity in our study (*P* = 0.072) (Fig. 3). Different quality scores between articles also did not account for the heterogeneity (*P* = 0.388). The presence or absence of adjustment for confounding factors in the articles also not the cause of heterogeneity (*P* = 0.729). The different regions and follow-up duration explained some of the heterogeneities (*P* = 0.035 and *P* = 0.014, respectively). However, the result of meta-regression indicated that the different regions had no effect on the pooled estimate of the HR (All *P* > 0.05), and study duration less than 10 years may be a source of heterogeneity (*P* = 0.035). The pooled estimate of the HR was 1.30 (95% CI: 1.12–1.52) in the articles with a follow-up duration longer than 15 years, compared with 1.33 (95% CI: 1.15–1.53) and 1.10 (95% CI: 1.05–1.15) in those with a follow-up duration between 10 and 15 years and less than 10 years, respectively.

### Sensitivity analysis and publication bias

Sensitivity analysis did not change the results of this meta-analysis. The Begg’s funnel plot was used to estimate the potential for publication bias (Supplementary Fig. 2). The result of Begg’s test (*P* = 0.001) indicated the possibility of publication bias in all-cause mortality. The trim-and-fill method was applied to test and adjust for publication bias, which conservatively hypothesized publication bias is the reason for funnel plot asymmetry. Some negative unpublished studies were used to produce a symmetrical funnel plot (Supplementary Fig. 2), which continued to reveal a statistically significant sex-specific association between T2D and all-cause mortality (HR: 1.08. (95% CI: 1.03–1.13)).

## Discussion

In this meta-analysis of 35 prospective cohort studies, which included data for more than 2 314 292 individuals and 254 038 all-cause mortality events, T2D was demonstrated as one of the risk factor for all-cause and CHD mortality in both women and men. Diabetes-related mortality was higher in women than men, and the effect of diabetes on all-cause and CHD mortality was 17 and 97% higher in women than men, respectively.

Similarly, the effect of type 1 diabetes on mortality was 37% higher in women than men (67). In a recent collaborative meta-analysis, diabetes was associated with all-cause mortality, and the relative risks were 1.59 in men and 2.00 in women, respectively (68). It is not clear why the rate of diabetes-related death was higher in women than in men, but several mechanisms could help to explain. One hypothesis is that the excess risk of mortality in women is due to the combined effect of greater deterioration and more prolonged exposure to cardiovascular risk profile during their prediabetic period (12, 67, 69). Due to their poorer glycemic control, women with diabetes had an overall greater cumulative lifetime exposure to hyperglycemia (12). Several studies have suggested that women with diabetes had higher BMI (70, 71, 72) and were more insulin resistant (72) than their men counterparts. And they also had significantly higher blood pressure and lipid levels than in men (12).

Research indicates that men with diabetes were diagnosed earlier than women (73), as the early symptoms of diabetes in man may be more likely to be recognized by physicians (74). Men with diabetes may receive better therapeutic interventions and more comprehensive care (75, 76, 77). Alternatively, sex differences in the management and treatment of diabetes may play a crucial role in the disparity in mortality rates between men and women (74). In addition, men with diabetes are more likely to use aspirin (78), which was proven to decrease the risk of stroke and myocardial infarction (79, 80). Furthermore, it has been reported that more diabetic men than women received recommended care processes (62 vs 58%) (77). Notably, even under the same treatment regimen, women were less likely than men to achieve treatment targets for controlling mortality risk factors (81, 82). Moreover, it has been reported that women were less likely to achieve glycemic targets with insulin glargine and exhibited significantly less reductions in fasting blood glucose levels (83). Previous research also observed that women were more likely to experience hypoglycemia during insulin treatment (84, 85). Therefore, differences in treatment and management may explain a large component of the excess risk associated with diabetes in women. In addition, women have less stroke risk factors compared with men (86), so the effect of adding one risk factor (such as diabetes) on women may be more serious.

The other potential mechanisms for sex-specific differences in mortality may result from the differences in biological factors. A recent study (87) suggested that diabetic women had higher levels of endogenous testosterone, which could predict incident CHD risk (88, 89). Women with diabetes also had a greater change in insulin resistance than men (90). Mansfield and colleagues also found sex-based differences in the level of coagulation and fibrinolysis in individuals with diabetes (91, 92) and reported that factor VII and plasminogen activator inhibitor 1 activity levels were significantly higher in women than in men, contributing to the increased cardiovascular risk. Furthermore, higher levels of adiponectin were associated with all-cause mortality in people with T2D (92, 93), and diabetic women were found to have higher levels of adiponectin (92).

The large sample size is one of the strengths of this meta-analysis. We are also the first study to estimate reliably the effect of T2D on CHD mortality risk among women in comparison with men. Additionally, the included studies were limited to prospective cohort studies, which eliminated the possible recall and selection bias. The subgroup and sensitivity analysis was used to explore the possible heterogeneity and ensure the reliability of the results. The trim-and-fill method was applied to adjust the potential publication bias. For the quality control of this meta-analysis, we also registered it at Prospero and performed the study in accordance with the PRISMA statement.

However, there were several limitations in our meta-analysis. Firstly, the standard definition of diabetes and confounding variables adjusted varied across studies, which may have resulted in inconsistent estimation of mortality risks. Secondly, the follow-up duration of T2D was not directly reported in some studies. Moreover, in most studies, diabetic status was mainly based on self-report or past medical history; therefore, there was a higher probability of underestimation of the number of patients with T2D. Although we performed a range of sensitivity analyses, we were also unable to explain most of the heterogeneity among the studies for the outcome of all-cause mortality. In addition, some articles lacked specific data on patient’s age; therefore, we could not perform age-specific subgroup analysis.

Taken together, we found that the relative effect of diabetes on all-cause and CHD mortality was significantly greater in women than in their men counterparts. For future, we should avoid sexual prejudice in diabetes, take all necessary steps to diagnose early and control risk factors comprehensively to guarantee the most suitable treatments in women patients. Besides, it is necessary to perform further studies to determine the actual mechanisms that account for sex-based difference in diabetes-related mortality risk.

## Supplementary Material

Supplementary Data

Supplementary Fig. 1

Supplementary Fig. 2

Supplementary Fig. 3

Supplementary Fig. 4

Supplementary Fig. 5

Supplementary Fig. 6

## Declaration of interest

The authors declare that there is no conflict of interest that could be perceived as prejudicing the impartiality of this study.

## Funding

The study is supported by Natural Science Foundation of Zhejiang Province (LY17H260002), K C Wong Magna Fund in Ningbo University, China Postdoctoral Science Foundation funded project (156458), Jiangsu Postdoctoral Science Foundation funded project (1601121B), Natural Science Foundation of Ningbo (2016A610169), Public welfare technology and policy science (soft science) application research of Zhejiang Province (2017C35006), Ningbo Scientific Innovation Team for Environmental Hazardous Factor Control and Prevention (2016C51001), Project of Science and Technology Innovation for College Students in Zhejiang Province (2018R405092), Sanming Project of Medicine in Shenzhen (SZSM201803080).

## Author contribution statement

L H, L L and D Y conceived the study, interpreted the data and drafted and critically revised the report. G X, J Y X and L W did the search, analyzed and interpreted the data and critically revised the report. J Z, X Z and L N Z critically revised the report. D D and F K participated in data collection, oversaw the data analysis and interpreted the data.
