# Supplementary material for: Risk of all-cause and CHD mortality in women versus men with type 2 diabetes: a systematic review and meta-analysis
Source: Eur J Endocrinol. 2019 Jan 21;180(4):243–55. doi: 10.1530/EJE-18-0792 (PMC6391911; doi:10.1530/EJE-18-0792)
Supplement: Supplementary Data [file supplementary_appendix_1.pdf]

Supplementary appendix 1

Search strategy on PubMed MEDLINE (www.ncbi.nlm.nih.gov) on Aug 7, 2018

(((((“Cardiovascular Diseases” [mesh] OR “mortality” [mesh] OR “death” [all fields] OR “heart disease” [all fields] OR “cerebrovascular disease” [all fields] OR “all-cause mortality” [all fields] OR “mortality” [all fields]))) AND ((“Diabetes Mellitus, Type 2” [Mesh] OR “type 2 diabetes” [all fields] OR “Adult-Onset Diabetes” [all fields] OR “Non-Insulin-Dependent Diabetes” [all fields]))) AND (((“men” [MeSH Terms] OR “men” [All Fields] OR “male” [MeSH Terms] OR “male” [All Fields]) AND (“women” [MeSH Terms] OR “women” [All Fields] OR “female” [MeSH Terms] OR “female” [All Fields])))) AND (((“Cohort Studies” [Mesh] OR “Prospective Studies” [Mesh] OR “Longitudinal Studies” [Mesh] OR “cohort” [All Fields] OR “prospective” [All Fields] OR “longitudinal” [All Fields])))

Newcastle-ottawa quality assessment scale for studies included in the meta-analysis

Selection

1) Representativeness of the exposed cohort
  a) truly representative of the average in the community (registries) *
  b) somewhat representative of the average in the community (cohort studies)
  c) selected group of users e.g. nurses, volunteers
  d) no description of the derivation of the cohort

2) Selection of the non-exposed cohort
  a) drawn from the same community as the exposed cohort *
  b) drawn from a different source
  c) no description of the derivation of the non-exposed cohort

3) Ascertainment of exposure
  a) secure record (age at diagnosis medically verified) *
  b) age at diagnosis based on start of medical treatment
  c) written self-report
  d) no description

4) Demonstration that outcome of interest was not present at start of study
  a) yes *
  b) no

Comparability

  1) Comparability of cohorts on the basis of the design or analysis
  a) study controls for age and sex in SMRs *
  b) study did not control for age and sex in SMRs (e.g. HRs or age-standardized SMR)

Outcome

  1) Assessment of outcome
  a) independent blind assessment *
  b) record linkage *

c) self-report

d) no description

2) Was follow-up long enough for outcomes to occur

a) yes (at least 10 years) *

b) no

3) Adequacy of follow up of cohorts

a) complete follow up - all subjects accounted for *

b) subjects lost to follow up unlikely to introduce bias > 10% follow up, or description provided of those lost *

c) follow up rate < 90% and no description of those lost

d) no statement
