# Supplementary figures and images for: Risk of all-cause and CHD mortality in women versus men with type 2 diabetes: a systematic review and meta-analysis
Source: Eur J Endocrinol. 2019 Jan 21;180(4):243–55. doi: 10.1530/EJE-18-0792 (PMC6391911; doi:10.1530/EJE-18-0792)

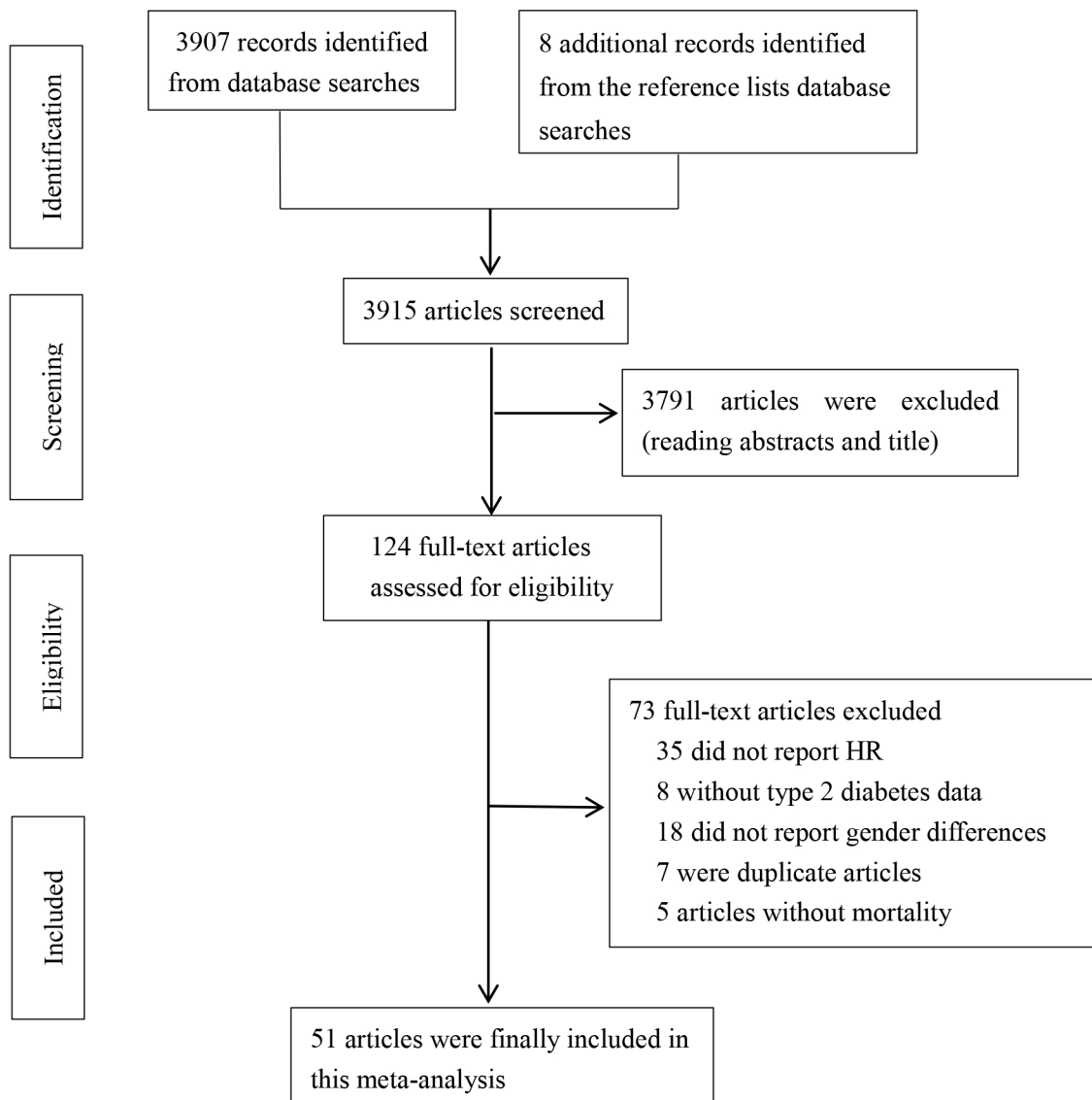

Supplement: Supplementary Fig. 1 [file supplementary_figure_1.pdf]

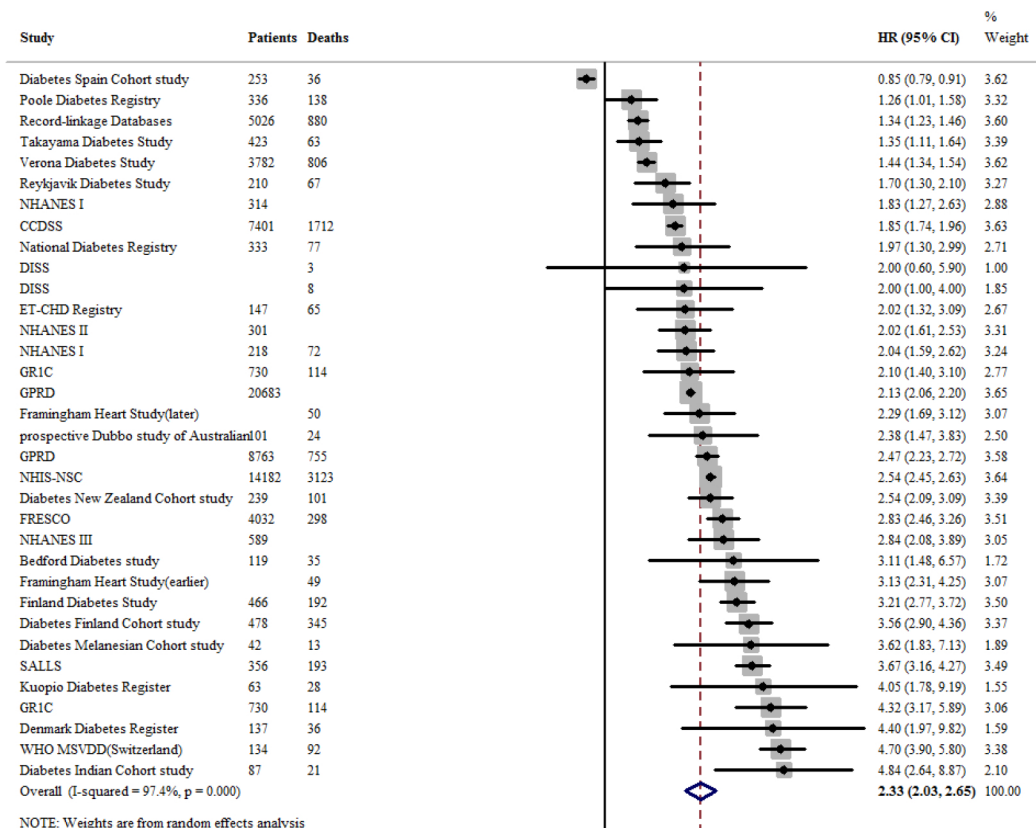

.102

Diabetes less Risky

1

Diabetes more Risky

9.82

Supplement: Supplementary Fig. 3 [file supplementary_figure_3.pdf]

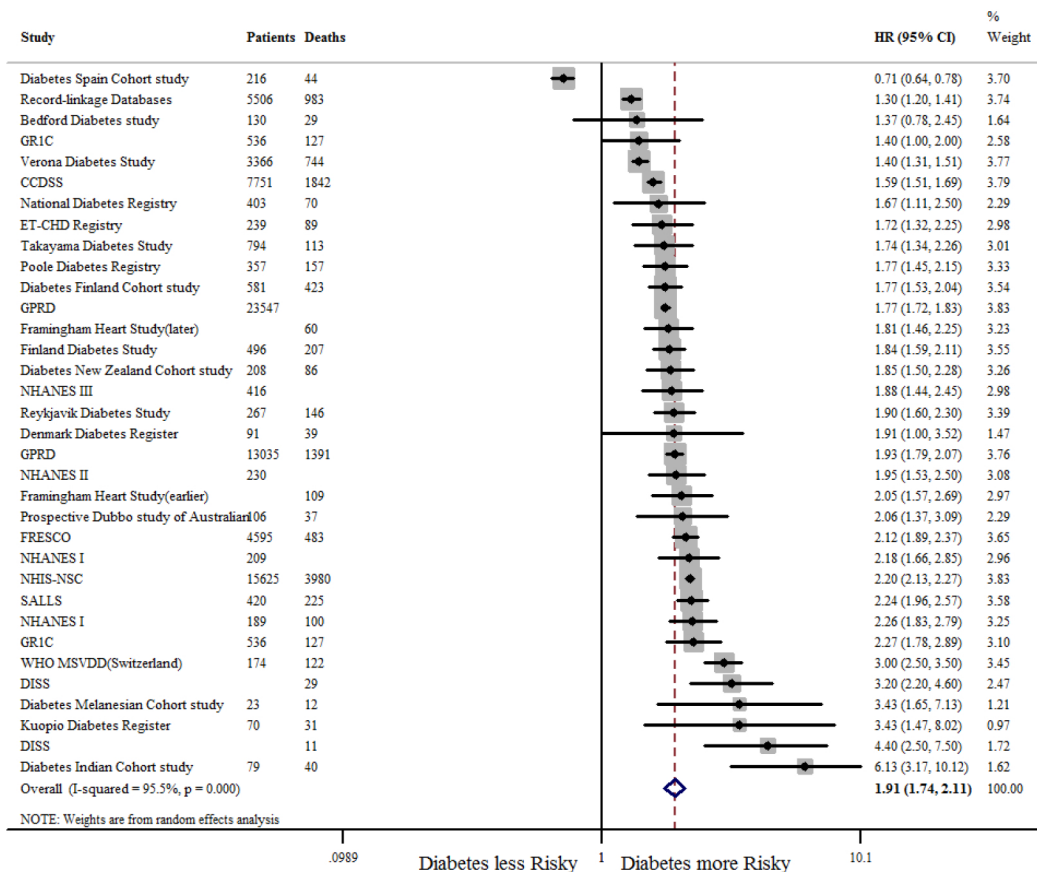

Supplement: Supplementary Fig. 4 [file supplementary_figure_4.pdf]

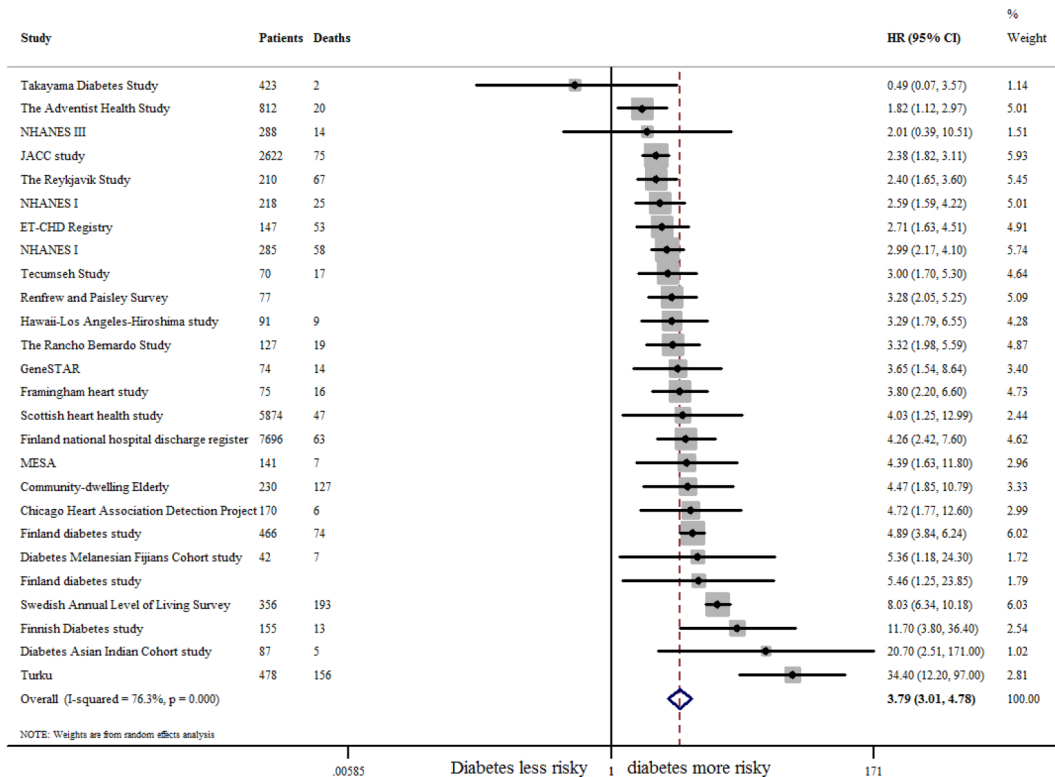

Supplement: Supplementary Fig. 5 [file supplementary_figure_5.pdf]

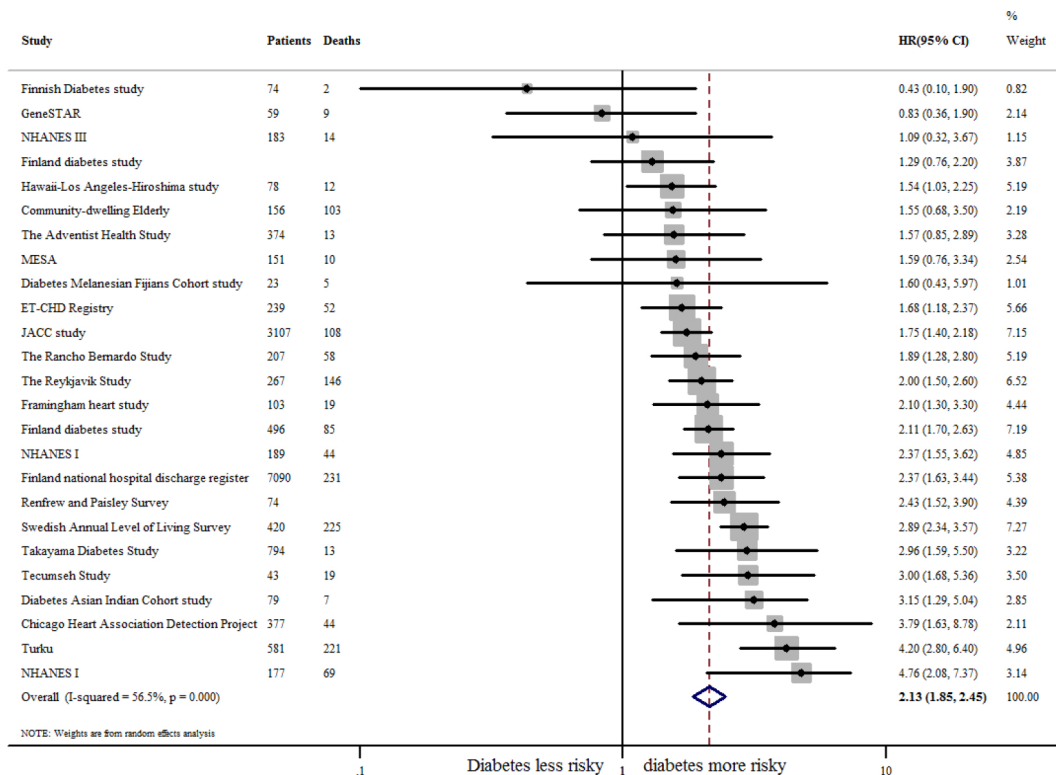

Supplement: Supplementary Fig. 6 [file supplementary_figure_6.pdf]
