# Supplementary material for: Risk of all-cause and CHD mortality in women versus men with type 2 diabetes: a systematic review and meta-analysis
Source: Eur J Endocrinol. 2019 Jan 21;180(4):243–55. doi: 10.1530/EJE-18-0792 (PMC6391911; doi:10.1530/EJE-18-0792)
Supplement: Supplementary Fig. 2 [file supplementary_figure_2.pdf]

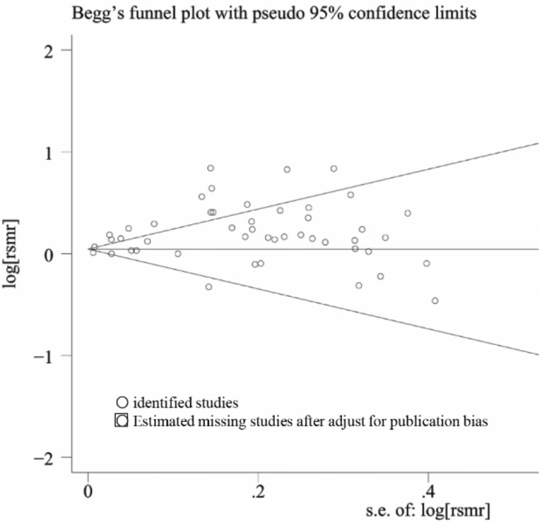

Begg's funnel plot with pseudo 95% confidence limits
2
1
0
−1
−2
log[rsmr]
identified studies
Estimated missing studies after adjust for publication bias
0
.2
.4
s.e. of: log[rsmr]

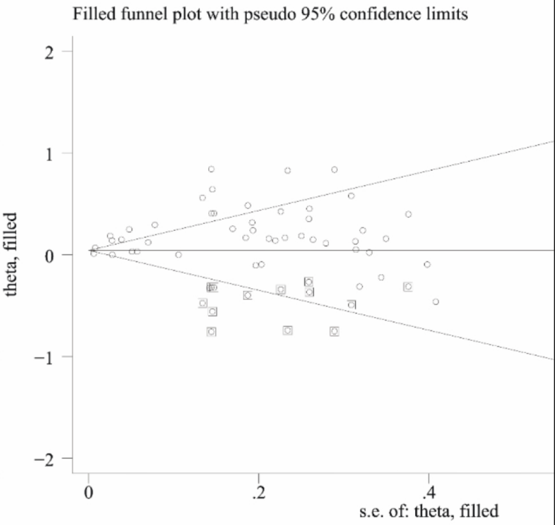

Filled funnel plot with pseudo 95% confidence limits
2
1
0
−1
−2
theta, filled
0
.2
.4
s.e. of: theta, filled
